# GENOMEPOP: A program to simulate genomes in populations

**DOI:** 10.1186/1471-2105-9-223

**Published:** 2008-04-30

**Authors:** Antonio Carvajal-Rodríguez

**Affiliations:** 1 Departamento de Bioquímica, Genética e Inmunología. Universidad de Vigo, 36310 Vigo, Spain

## Abstract

**Background:**

There are several situations in population biology research where simulating DNA sequences is useful. Simulation of biological populations under different evolutionary genetic models can be undertaken using backward or forward strategies. Backward simulations, also called coalescent-based simulations, are computationally efficient. The reason is that they are based on the history of lineages with surviving offspring in the current population. On the contrary, forward simulations are less efficient because the entire population is simulated from past to present. However, the coalescent framework imposes some limitations that forward simulation does not. Hence, there is an increasing interest in forward population genetic simulation and efficient new tools have been developed recently. Software tools that allow efficient simulation of large DNA fragments under complex evolutionary models will be very helpful when trying to better understand the trace left on the DNA by the different interacting evolutionary forces. Here I will introduce GenomePop, a forward simulation program that fulfills the above requirements. The use of the program is demonstrated by studying the impact of intracodon recombination on global and site-specific *dN/dS *estimation.

**Results:**

I have developed algorithms and written software to efficiently simulate, forward in time, different Markovian nucleotide or codon models of DNA mutation. Such models can be combined with recombination, at inter and intra codon levels, fitness-based selection and complex demographic scenarios.

**Conclusion:**

GenomePop has many interesting characteristics for simulating SNPs or DNA sequences under complex evolutionary and demographic models. These features make it unique with respect to other simulation tools. Namely, the possibility of forward simulation under General Time Reversible (GTR) mutation or GTR×MG94 codon models with intra-codon recombination, arbitrary, user-defined, migration patterns, diploid or haploid models, constant or variable population sizes, etc. It also allows simulation of fitness-based selection under different distributions of mutational effects. Under the 2-allele model it allows the simulation of recombination hot-spots, the definition of different frequencies in different populations, etc. GenomePop can also manage large DNA fragments. In addition, it has a scaling option to save computation time when simulating large sequences and population sizes under complex demographic and evolutionary situations. These and many other features are detailed in its web page [1].

## Background

There are several situations in population biology research where simulation of DNA sequences is useful. Simulations have been used to for hypothesis testing [2-4], to study the impact of differing demographic scenarios on patterns of human diversity [5], or to simulate the evolution of complex diseases in human populations [6,7]. In addition, population simulation of genetic datasets is also used to estimate population parameters [8-10].

One of the most exciting research areas in the current context of population genetics is the HapMap project. Knowledge about patterns of linkage disequilibrium (LD) in humans is very important from a genomic point of view. The existence of linkage or haplotype blocks [11] or, at least, networks of SNPs in high LD [12], will facilitate the assembly of human genome haplotype maps [13-15] that will enormously improve, among other things, the efficiency of disease gene mapping. It seems that these blocks are mainly defined by recombination hot spots [16,17], but haplotype blocks can also be generated by genetic drift in regions of uniform recombination if rates is low enough [18]. We have now growing empirical knowledge about haplotype block and tagSNP diversity, but less is known about the effect of population demographic history. Though important work has been undertaken in the application of population genetics to LD mapping [19-22] and its relevance to human populations [23-25], we still have an incomplete understanding of how the combined effect of genetic drift, mutation, recombination and migration, affect LD and tagSNP patterns, although it is known that they do [26]. Moreover, recombination is an important evolutionary process to understand how genetic diversity is generated and maintained in populations. Jointly with positive selection, recombination allows for very high rates of evolution [27]. However, the impact of recombination is dependent on other forces, such as selection and demography. Developing tools that allow simultaneous simulation of natural selection, recombination and complex demographic patterns will be of great help in trying to better understand the trace left on the DNA by the different interacting evolutionary forces.

Simulation of biological populations under different evolutionary genetic models can be done following backward or forward strategies. Backward simulations, also called coalescent-based simulations, are computationally very efficient because they are based on the history of lineages with surviving offspring in the current population and ignore all individuals that are not ancestral to the present-day population [20]. Hence, coalescent is a sample-based theory relevant to the study of population samples and DNA sequence data. From its beginnings, the basic coalescent has been extended in several useful ways. For example, to include structured population models [28-32], changing population size [33-35], recombination [36,37] and selection [38-43].

On the contrary, forward simulations are less efficient because the entire population is simulated from past to present. However, the coalescent framework imposes some limitations that forward simulation does not. The first of these is the same feature that causes its efficiency, namely, the coalescent does not keep track of the complete ancestral information i.e. only takes into account ancestries that survived to form the present-day sample. Thus, if the interest is focused on the evolutionary process itself, rather than on its outcome, forward simulations should be preferred [44]. Second, coalescent simulations are complicated by simple genetic forces such as selection, and although different evolutionary scenarios have been incorporated (see above) it is still difficult to implement models incorporating complex evolutionary situations with selection, variable population size, recombination, complex mating schemes, and so on. In fact, we can only simulate limited forms of recombination and selection under the coalescent. It is known that recombination has a major impact for detecting positive natural selection [45,46]. Shriner *et al *studied the impact of recombination under a neutral model. Anisimova *et al *studied the recombination effect under a coalescent codon-based model i.e. the unit of change was the codon instead of the nucleotide. In the latter case, recombination was not simulated at the intracodon level. Therefore, we still ignore the importance of intracodon recombination under a given codon-based model. Moreover, coalescent methods cannot yet simulate realistic samples of complex human diseases [6]. Indeed, when simulating non-neutral scenarios and/or complex models under the coalescent, much of its computational efficiency is lost (however, see recent work by Marjoram [47] and Liang [48]). Furthermore, the coalescent model is based on specific limiting values and relationships between some important parameters [49]. Hence, there is increasing interest in forward population genetic simulation and new efficient tools have been recently developed [50-52]. Therefore, a program that allows the simulation forward in time, of different Markovian nucleotide or codon models of DNA mutation combined with recombination, at inter and intra codon levels, fitness-based selection and complex demographic scenarios, will be of great interest. Here I will introduce the program GenomePop that fulfills the mentioned requirements.

## Implementation

GenomePop uses a simple and efficient algorithm to perform forward simulation of populations and/or genomes. The basic idea considers an individual as the differences (mutations) between this individual and a reference or consensus genotype. Thus, each individual is no longer represented by its complete sequence or genotype but by the mutations it carries with respect to the consensus. A more detailed explanation of the algorithm is provided at the program web page. Taking advantage of the efficiency of this approach, GenomePop can simulate, forward in time, DNA sequences under specific Markov models. The program allows the simulation of recombination under both nucleotide and codon models of evolution, providing a way to simulate recombination at inter and intracodon levels under codon models. It also permits arbitrary migration models, simulation of SNPs, recombination hot-spots, fitness-based selection and many other features that are detailed in the program web-page. GenomePop has different output formats as GenePop for SNPs and Phylip or Nexus for DNA sequences.

### Markov models of DNA mutation

Markov processes are used in molecular evolution to describe the change between nucleotides, aminoacids or codons over evolutionary time. Usually, time is measured as the number of substitutions because molecular sequence data does not allow the separate estimation of the rate and the time, but only of their product [53]. In the context of forward simulation we are not interested in the transition after an arbitrary time *t *(branch length) but just in the transition from a nucleotide or codon to another, given that a mutation occurs. An advantage of this approach is that we need to compute the transition matrix just once at the beginning of the evolutionary process. Therefore, consider a given instantaneous substitution rate matrix *Q*, which allows for a complete definition of any Markovian substitution model [53], the matrix *M *= -*qQ *+ *I *is the conditional transition matrix to go from *i *to *j *provided that a substitution occurs, where *q *= diagonal (1/*q*_*i*_) and *I *is the identity matrix [54]. Then, given an instantaneous substitution matrix *Q*, estimated for example using PAUP [55] or Hyphy [56] programs, we can obtain the corresponding transition matrix *M *that can be used to produce the necessary mutation process in a forward in time evolutionary model.

### Biological models

There are two basic biological models implemented in GenomePop, namely "viral" and "non-viral". The only difference that distinguishes them is just that in the viral model the initial sequences are different in each population, as the different viruses infect different individuals. Thus, the user can define a viral model indicating the percentage of sequence identity (0–100) between the sequences of the distinct populations. By default the sequence identity is zero i.e. the sequences at each population are randomly settled. In the non-viral model the initial sequence is the same for every population (identity of 100%).

### DNA models, recombination and selection

There are different DNA models implemented in GenomePop (Table 1). In any of them, the user can decide to allow recurrent mutation, i.e. multiple site hits or not. Models can be haploid or diploid. Population size can be constant or variable. In the four-allele models, the sequences can be generated by the program or provided by the user. In the case of the 2-allele model (SNPs) just one or several chromosomes can be considered. In this same model, recombination can be constant or a hot spot recombination model can be defined. In the latter, the recombination rate *r *is per haploid region and generation. If no hot spots are defined, the expected number of recombination events between any two sites *i *and *j *will be 2*rd*_ij_/(*L*-1) where *d*_ij _is the implied region length and *L *is the chromosome length. The number of recombination events between the two chromosome extremes 0 and *L *-1 will be 2*rd*_ij_/(*L*-1) = 2*r*. In GenomePop, the effect of natural selection can be modelled in two different ways: 1) by its effects on the *dN/dS *ratio i.e. by defining a codon model, and 2) via the fitness effect of mutation on specific loci. The user can run either of two models. The codon model option runs a MG94 codon model [57] with a given *dN/dS *combined with any defined nucleotide model. This model of codon evolution will be implemented by the instantaneous rate matrix to go from codon *i *to *j*. That is, *Q*_ij _= *θ*_*mn*_*kπ*_*n *_where *θ*_*mn *_accounts for biased nucleotide, *m *to *n *substitutions; *k *= 1 or ω for synonymous or nonsynonymous mutation rates respectively and *π*_*n *_is the equilibrium frequency of the target nucleotide. This corresponds to the MG94 model [57] with the restriction of α = 1. Nucleotide equilibrium frequencies are used instead of codon frequencies. To simulate a given *dN*/*dS *we simply set ω = *dN*/*dS*. Alternatively, the user can set the codon model option to false (default option) and define specific sites under directional selection with a given selective coefficient which will apply when a mutation occurs at such site. The user can also force all sites to undergo selection. The selection coefficient, *s*, can be constant or sampled from a gamma distribution with user-defined shape parameter β and scale parameter β/*s*. The β parameter allows for modelling of the fitness effects distribution, e.g. a low value of β (0.1) will sample many mutations with low effect and few with high. A β parameter of 1 corresponds to the exponential distribution. If we set β to 0 then a constant effect model is applied. Moreover, GenomePop permits the combination of both kinds of models of selection, codon and fitness-based, though the biological meaning of such a mixture is not clear.

**Table 1:** GenomePop DNA models

DNA Model	GenomePop Notation	Output format	Recombination	Selective sites
2 allele	JC2	Genepop	Hot spots	Yes
Jukes Cantor	JC4	Phylip/Nexus	Constant	Yes
GTR	GTR	Phylip/Nexus	Constant	Yes
MG94 × (JC/GTR)	Codon true	Phylip/Nexus	Constant	Yes

GTR: General Time Reversible Model [63]. MG94: Muse and Gaut [57] codon model.

### Migration models

**Figure 1:**
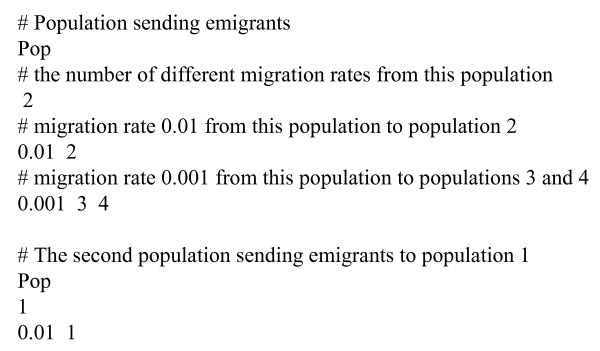
Example of a user-defined migration model.

Two basic migration schemes, island model and one-dimensional stepping stone, are pre-defined in GenomePop. However, the user can define any migration model of interest (Figure 1). To do this, set the flow model to 'user' in the standard input file and then just introduce a scheme similar to that of Figure 1 in a file called MigrationModel.txt. In this file, the lines beginning with '#' are comments. To indicate how individuals will migrate from a given population just begin the line with the word "pop". The order of appearance of each population in the file will correspond with its index i.e. the first population that appear is the population number one, etc. The number below "pop" refers to the migration level, i.e. the number of different migration rates defined from this population. The next line should begin with a migration rate (between 0 and 1) followed, in the same line, by the target population(s). We should have as many of these kinds of lines as the migration level indicates, i.e. if the migration level is 2 we should have two lines beginning with a migration rate. More detailed explanation and specific examples are given in the program web page.

### Scaling

Clearly, the more complex the model defined, the slower the simulation. To avoid high computation times, GenomePop incorporates a scaling option based on the fact that, under neutral models, we can scale the population size *N *and the time *t*, provided the consequent correction to the mutation (μ), migration (*m*) and recombination (*r*) rates holds the corresponding compound products *Nμ*, *Nr*, *Nm*, etc., constant.

## Results

### Input file

**Figure 2:**
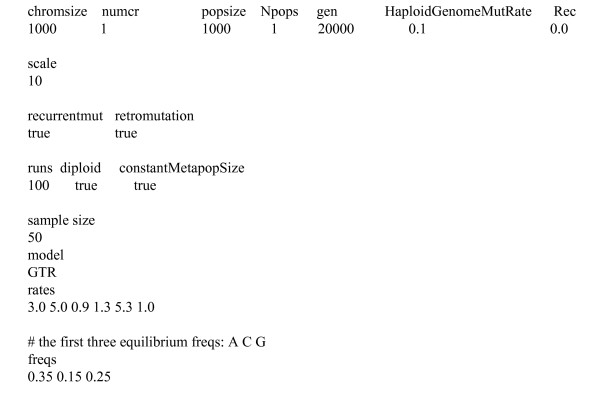
Input file to generate 100 datasets under a GTR model.

The input file should be called GenomePopInput.txt. In this file, lines beginning with '#' are comments and will be ignored. In Figure 2 we can see an example of an input file. Note that the input is flexible, i.e. the minimum input for GenomePop to work appropriately corresponds to the first line and the values below it. This line must begin with the identifier 'chromsize' and the line below with the corresponding desired values. Note that, in lines with identifiers, only the first word matters for the program.

Thus, the input in Figure 2 generates 100 datasets under a GTR model with substitution rates typical for HIV [58]. Both recurrent and retromutation are allowed. The system will evolve 1 chromosome of 1 Kb under the given model over 20,000 generations. As can be seen in Figure 2, a scaling of 10 was used, which implies that both, population size and the number of generations, was divided by 10 and mutation was multiplied by the same factor. A more exhaustive explanation of the input facilities of GenomePop is provided at the program web page.

### Example and validation of the Markov mutation method

For each obtained dataset from the input in Figure 2, the best-fit model of nucleotide substitution under the Akaike information criteria (AIC) was estimated with Modeltest v3.6 [59], using maximum likelihood (ML) estimates from PAUP* [55]. The percentage of correct model estimation (GTR) was 97% although some datasets, about 29%, were also assigned invariable sites or rate heterogeneity among sites. The substitution pattern and equilibrium frequencies were correctly estimated.

### Examples and validation of other general features

**Figure 3:**
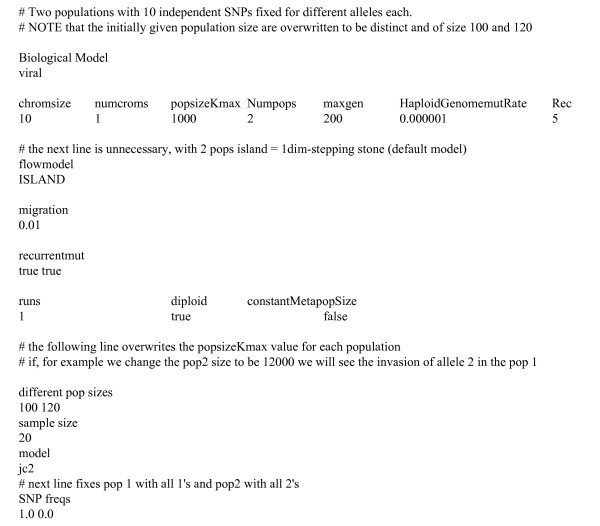
Input file to generate 10 independent SNPs at different frequencies in different populations.

As GenomePop has many different features and models it is difficult to validate every possibility or circumstance. However, strong effort has been made to validate the program as thoroughly as possible. For example, both unscaled and scaled simulations were performed under a Jukes-Cantor model with diversity θ = 4*Nμ *= 0.004 over 10^4 ^generations and then θ was estimated using the finite-sites correction of Watterson θ [60]. The accuracy was quite good, obtaining estimates of 0.0043 ± 0.00015 and 0.0037 ± 0.00016 for the unscaled and scaled cases respectively. Recombination was also tested by evolving datasets for 6*N *generations under a Jukes-Cantor 4-allele model with different values for the parameter ρ = 4*NrL*, where *N *is population size, *r *is recombination rate per site and *L *is the DNA sequence length (the corresponding parameter in GenomePop is 'Rec' = *r *× *L*). Namely, we ran cases with ρ equal to 0, 50 and 100. Recombination was then accurately estimated using the program Kpairwise [58]. GenomePop allows also studying 2-allele SNPs at different frequencies in different populations. In Figure 3 we define a 2-allele model (JC2) with different initial composition at each population (viral model) and 10 independent SNPs (recombination 'Rec' = 10 × 0.5 = 5). The populations have different sizes (100 and 120) and migration occurs under the island model. Note that when defining different population sizes, the original population size provided in the 'chromsize' line under the 'popsizeKmax' identifier is overwritten.

We ran this example over 200 generations and then analyze the output with the GenePop 4.0 program [61]. As expected the SNPs were detected as independent. We then changed the value of recombination to 0 ('Rec' = 0) and then GenePop 4.0 tell us that the 10 SNPs are linked, as expected. Note the many possibilities that the program provides in the context of studying SNPs under complex evolutionary situations. We can define any number of populations under any user-defined migration model. We can set any number of SNPs with the desired linkage relationships. The SNPs can be set at distinct initial frequencies in the different populations, for example, 'SNPfreqs' at 1.0 and 0.0 defines the first population with allele 1 fixed and the second with allele 2 fixed.

### Impact of recombination on estimation of positive selection

We performed a simple experiment to test the impact of recombination on *dN*/*dS *estimation. We ran 50 replicates, with and without population recombination per gene, 4*Nr *= 40 and 0, respectively. The runs were performed under a MG94 × JC model both with *dN*/*dS *= 1 and *dN*/*dS *= 2.5 evolving 333 codons for 10*N *generations with an effective population size of *N *= 10^3 ^to get samples of 20 sequences. The *dN*/*dS *ratio was estimated with the FEL (Fixed effects Likelihood) model of Hyphy [62] which computes global and site by site *dN/dS *ratio. A *p *value of 0.1 was used to infer sites under positive selection. As can be seen in Table 2 a *dN/dS *of 2.5 provokes the detection of some sites under positive selection (1 or 2, not shown) in only 30% of the replicates (NSS = 0.3 in Table 2). Furthermore in the strictly neutral case (*dN/dS *= 1), one positive selected site was assigned in 10% of the replicates as expected given the *p *value used. If we correct by this 10% of false positive tests then positive selected sites were detected only in 20% of the replicates under a *dN/dS *value of 2.5 and no recombination. This is in agreement with the conservative nature of the FEL method [62]. Also noteworthy is that recombination had no impact on global *dN*/*dS *estimation but had important effects on the number of sites detected under positive selection as is evident upon inspecting Table 2. It seems also that the effect of intracodon recombination is negligible. Interestingly, it appears that the effect of recombination is somewhat higher under non-neutral *dN/dS *than in the neutral case. The impact of recombination on positive selection detection has already been studied [45,46]. However, as far as we know, the comparison of the impact of recombination under neutral or positve *dN/dS *jointly with the effect of intracodon recombination has never been studied before. The significance of this effect should be studied with more replicates and cases, which is out of the scope of the present work.

**Table 2:** Impact of recombination on *dN/dS *estimation under a Jukes Cantor model.

4*Nr*	Expected *ω*	Estimated *ω*	NPSS
0	1	1.02 ± 0.03	0.1 ± 0.05
40	1	1.06 ± 0.04	9.9 ± 0.56
40 ncb	1	1.01 ± 0.03	8.8 ± 0.49
0	2.5	2.62± 0.12	0.3 ± 0.07
40	2.5	2.57± 0.11	13.1 ± 0.77
40 ncb	2.5	2.58± 0.13	12.7 ± 0.65

*N*: Population size. *r *= Recombination rate per gene. *ω *= *dN*/*dS*. NPSS: Average number of positive selection sites. ncb: no codon break allowed.

## Conclusion

GenomePop has interesting characteristics for simulating SNPs or DNA sequences under complex models of evolution and demography. These features make it unique with respect to other simulation tools. Namely, the possibility of forward simulation under GTR mutation or GTR × MG94 codon models with intra-codon recombination, simulation of any user-defined migration pattern, diploid or haploid models, constant or variable population sizes, fitness-based selection, etc. Under the 2-allele model it allows the simulation of recombination hot-spots, the definition of different frequencies in different populations, etc. GenomePop can also manage large DNA fragments and has a scaling option to save computation time when simulating large sequences or population sizes under complex demographic and evolutionary situations. It has many other features that are detailed in the web page [1].

## Availability and requirements

**Project name: **GenomePop v. 1.0

**Project home page: **

**Operating system(s): **Windows and Linux (the source will be provided to compile for Mac)

**Programming language: **C++

**License: **GNU GPL.

## Authors' contributions

AC-R had the original idea for the work, designed and implemented the algorithms and wrote the manuscript.
